# What are the mechanisms of effect of group antenatal care? A systematic realist review and synthesis of the literature

**DOI:** 10.1186/s12884-024-06792-6

**Published:** 2024-10-01

**Authors:** Anita Mehay, Giordana Da Motta, Louise Hunter, Juliet Rayment, Meg Wiggins, Penny Haora, Christine McCourt, Angela Harden

**Affiliations:** 1 School of Health and Psychological Sciences, City, University of London, Myddelton Street, London, EC1R 1UW UK; 2 Oxford Brookes University, Oxford, UK; 3 University College London, London, UK; 4 University of Queensland, Brisbane, Australia

**Keywords:** Group care, Group antenatal care, CenteringPregnancy, Realist review, Maternity, Mechanisms

## Abstract

**Background:**

There is growing interest in the benefits of group models of antenatal care. Although clinical reviews exist, there have been few reviews that focus on the mechanisms of effect of this model.

**Methods:**

We conducted a realist review using a systematic approach incorporating all data types (including non-research and audiovisual media), with synthesis along Context-Intervention-Mechanism-Outcome (CIMO) configurations.

**Results:**

A wide range of sources were identified, yielding 100 relevant sources in total (89 written and 11 audiovisual). Overall, there was no clear pattern of ‘what works for whom, in what circumstances’ although some studies have identified clinical benefits for those with more vulnerability or who are typically underserved by standard care. Findings revealed six interlinking mechanisms, including: social support, peer learning, active participation in health, health education and satisfaction or engagement with care. A further, relatively under-developed theory related to impact on professional practice. An overarching mechanism of empowerment featured across most studies but there was variation in how this was collectively or individually conceptualised and applied.

**Conclusions:**

Mechanisms of effect are amplified in contexts where inequalities in access and delivery of care exist, but poor reporting of populations and contexts limited fuller exploration. We recommend future studies provide detailed descriptions of the population groups involved and that they give full consideration to theoretical underpinnings and contextual factors.

**Registration:**

The protocol for this realist review was registered in the International Prospective Register of Systematic Reviews (PROSPERO CRD42016036768).

**Supplementary Information:**

The online version contains supplementary material available at 10.1186/s12884-024-06792-6.

## Background

Antenatal care (ANC) is considered an integral component of maternity care and can make a vital contribution to improving health outcomes and reducing inequalities [1]. According to the UK’s National Institute for Clinical Excellence (NICE), pregnancy care should be woman-centred and enable informed decision making [2]. Some women are well prepared for the challenges brought on by the journey through this major life event, but many experience significant barriers to enabling optimal care for themselves and their babies [3]. There is growing evidence to suggest that care should be tailored to meet the diverse needs of women and birthing people, within sometimes complex social situations [4] but evidence on its implementation is scarce [3]. Many women report being overwhelmed with so much information and that care is not person-centred, particularly in hospital settings [5]. Within the context of hospital maternity services, in many countries, antenatal care is fragmented, leaving women feeling like ‘a number’ [6] with screening tests dominating antenatal appointments [7]. For many people from socially and ethnically diverse groups, the political, policy, clinical and philosophical contexts of maternity services make engagement with care challenging [8], leading to poorer maternity outcomes [9, 10]. Pregnant women (and their partners) are sometimes offered antenatal education classes. However, access and provision is inequitable across the UK and many do not attend due to cost and/or other constraints. Classes are considered important for providing information and facilitating social support, which is known to be important for short- and long-term wellbeing [11]. However, there is no consistent evidence that standard (didactic) antenatal education improves birth and parenthood outcomes and/or experiences [12].

A recent UK enquiry into maternal and child health highlighted the significantly higher mortality rates among women and babies from minority ethnic groups and those affected by social or economic deprivation and identified sub-optimal care experiences as a contributing factor [10]. Systemic, structural, and institutional factors can produce these health disparities and expose a pattern whereby women from socially and ethnically diverse groups receive inadequate maternity care. Frequently, there are multiple forms of intersecting inequalities which compound and create challenges and disadvantages based on numerous factors [13].

### Existing evidence on group antenatal care

Ensuring quality, equitable maternity care requires the development and evaluation of new care models and, where appropriate, scale-up and replication for maximum population health impact. Quality maternity care should incorporate medical checks, effective health information sharing, social support, and cultural safety for all women, to enable participation in timely and comprehensive care seeking. Satisfying and optimal care and outcomes may be supported with such holistic ANC models.

Group ANC is a care model combining elements of clinical assessment and learning with the aim of facilitating social connections [14]. One of the most established models is ‘Centering Pregnancy’, developed by a midwife in the US to tailor care to the needs of socially disadvantaged communities who experience poorer access and care quality [15]. Centering Pregnancy combines clinical checks with information sharing and is typically provided by the same two midwives facilitating a group of around 8–10 pregnant women. The first part of each session involves ‘self-care activities’ (individuals are taught to test their own blood pressure and urine) followed by one-to-one individual health checks with a midwife on a mat in the group space, to one side of the ‘Circle’ while other women had a group discussion facilitated by the second midwife. These clinical checks included: palpation, auscultation and fundal height measurement; discussion about blood pressure, urine, scan and blood results; and questions about foetal movements, personal concerns, mental health and domestic violence. Any general queries are discussed in the group, allowing the individual checks to remain brief (around 3–5 min). The model was developed in response to recognition of the importance of social support during pregnancy and the transition to parenthood and known limitations of didactic approaches to teaching and learning. Furthermore, women are not viewed as passive recipients of care, but are encouraged to make informed decisions, provide informed consent (or refusal), and to take an active role in their care to attain the best outcomes for themselves and their babies. This is facilitated by the longer time allocated for each session (typically 90–120 minutes compared with 20–30 in individual care) and continuity of facilitators and participants.

A 2015 Cochrane review of experimental studies concluded there is not yet sufficient evidence on group ANC to draw strong inferences about clinical outcomes [16] and a later systematic review of randomised controlled trials and cohort studies did not find significant differences in clinical outcomes [17]. However, a review focused on outcomes for women categorised as higher risk showed more variable effects, with greater benefits for specific groups including adolescents and African American women [18]. Group ANC is a complex, person-centred intervention, therefore it cannot be assumed that benefits identified in one study or setting will be scaled and replicated in others [19]; evaluations need to take account of practice variations and local contexts, including beliefs and views of local health professionals and of service users [20]. Group ANC also combines different components (i.e., continuity of midwifery carer, social support and enquiry-based learning) which in themselves may have different explanatory theories of effect. Emerging evidence suggests that empowerment and support are core principles of group ANC, which yields benefits for women in contexts with inequalities in access and delivery of care [21]. However, there are different theoretical perspectives to understanding the mechanisms of empowerment [22] and within group ANC, the concept is still under-theorised and poorly understood. Increasing our analytical understanding of the theoretical propositions that underpin group ANC, the ‘ingredients’ of the model, will help to explain any effects and the role of context, to support further developments of the model and inform scaleup and replication/adaptation. This calls for an approach rooted in critical realism [23] to better understand the underlying causal mechanisms and the interplay between observable and hidden mechanisms shaping how group ANC might work for particular groups and within different contexts.

### Realist synthesis approach

Realist synthesis is an approach to systematic review which focuses on identifying and testing potential context-intervention-mechanism-outcome configurations to develop theoretical and substantive understanding of how an intervention works, for whom and in what circumstances [24, 25]. It challenges positivist models of systematic review by positing that complex interventions do not ‘work’ in an ‘a-contextual’ and standardised fashion, replicable once subjected to rigorous evaluation. Instead, mechanisms of effect are produced by the ways in which interventions are interpreted, implemented and enacted, in particular environments and by people who may actively shape them [26]. In order to develop an appropriate experimental study, therefore, we identified a need to clearly understand potential mechanisms of effect of this care model and to develop a context-sensitive model which includes a core set of components around which local implementation would vary [27]. In this sense, realist reviews seek to provide explanations rather than measure outcomes.

This review was developed as part of a broader research programme, the REACH Pregnancy Programme [28], which sought to develop, implement and evaluate a bespoke model of group ANC (called ‘Pregnancy Circles’) for a socially and ethnically ‘superdiverse’ community [29]. The primary aim of this realist review was to articulate both implicit and explicit theories of action and key principles of group ANC (see protocol [30]). Secondary aims were to synthesise the findings/methods of the sources under review in relation to maternal and newborn health and wellbeing, and health services/service provider outcomes. The specific objectives were to:

1. Identify and review relevant research on/reports of implementation of group ANC models.
2. Articulate theories informing the models evaluated.
3. Identify the context and mechanisms of change in models already evaluated, recognising the likely complexity.
4. Synthesise and develop a set of core principles to inform the design and development of an intervention model tailored for a UK context named ‘Pregnancy Circles’.
5. Inform the preparations for implementing and testing the model in a planned multi-centre RCT.
6. Synthesise the findings of the range of the studies/sources on the subject.

## Methods

This review was conducted following the RAMESES guidelines for realist synthesis, and the PRISMA guidelines for systematic reviews [31, 32].

### Eligibility criteria

We envisioned different sources would contribute different context, intervention, mechanisms and outcome (‘CIMO’) insights (with some containing several data types). We therefore sought to mine for theoretical and empirical data in a wide range of media, including clinical trials, qualitative studies, reviews, reports, commentaries and videos. We included sources describing reviews as background information to provide theoretical insights; only sources describing primary research were accessed for data extraction and analysis. Non-research sources (e.g. opinion pieces, audio-visuals) were also included as these may highlight theoretical propositions underlying model development and implementation.

Inclusion criteria:

1. All sources related to any outcomes of an ANC model, or ANC and postnatal care that include participants meeting in a group (at least 4 women).
2. All sources related to the process or implementation of an ANC model that includes women meeting in a group (at least 4 women).
3. All sources related to experiences of an ANC model that includes women meeting in a group (at least 4 women) (professionals’ or users’ experiences).
4. All national/country contexts.

Exclusion criteria:

1. Groups that do not include ANC.
2. Groups provided outside NHS/mainstream health care (e.g., by charity groups).
3. Groups that provide speciality rather than routine care (e.g., obesity ‘clinic’).
4. One-off groups.
5. Groups not including clinical care (e.g., classes only).
6. Groups not involving any health professional input (e.g., peer-led groups).

Following data extraction, a further exclusion was applied prior to analysis:

7) Sources relating to opinions and experiences without relevant CIMO data.

### Study selection

Database searches were conducted in MEDLINE, PsychINFO, EMbase, Maternity and Infant Care, Web of Science, Cochrane library (Cochrane Central Register of Controlled Trials [CENTRAL] and Database of Systematic Reviews) (see Appendix 1 for search terms used). No language restrictions were imposed for the initial search. Sources published from and including January 1980 to March 2015 were eligible for inclusion. Grey literature was sought in sources including OpenGrey, GreySource, internal reports and non-peer reviewed journals such as Midwifery Digest). Reference chaining was undertaken on all relevant sources retrieved, and forward and back-citation searches conducted using Google Scholar. Searches were also undertaken in relevant websites such as the Centering Healthcare Institute Inc., Association for Improvements in Maternity Services, National Childbirth Trust, and Local Supervising Authority Midwifery Officers Forum. As we aimed to include audio visual media, we also searched YouTube and internet search engines (for example, Google) using key terms.

EPPI-Reviewer version 4 was utilised for data/review management. Titles and abstracts of written sources retrieved were first independently double screened by two researchers with any differences resolved through discussion or deferred to full text assessment. Full texts of included written sources were then double screened by two researchers, and any disagreements adjudicated by a third person. Audio visual media sources were screened by one reviewer using the same criteria. Realist reviewing is complex and time-consuming so following the analysis, the search was updated in April 2019 to identify any additional sources or insights (from the period of April 2015 - April 2019). These were screened and read analytically by two reviewers to identify whether any new themes should be added, or existing themes modified in light of new literature.

### Data extraction and management

We developed data extraction proformas to draw out data (i.e., descriptive notes, ideas and annotations or excerpts) around the terms of what works, for whom, in what circumstances. The extracted data were then interrogated by sub-teams of researchers to answer the research aims specifically relating to:

- **What works**,** and for whom?** Outcomes measured in each study were collated and compared by study population, to determine whether they were more or less successful with different groups of women (for example, vulnerable, young or socio-economically deprived groups).
- **How?** Are any explicit theoretical claims made about how the intervention might or did achieve the intended or experienced outcomes? What can be gathered implicitly regarding theories of how the intervention might or did work?
- **In what circumstances?** How does context at a strategic, institutional, inter-personal and individual level disrupt or support the implementation or delivery of the intervention?

The analysis was conducted inductively, with no initial attempt to impose a preconceived framework, theory or theories onto the data unless deemed appropriate. As part of the reflective process team members formulated their own logic models prior to analysis, to make their own ‘theories’ explicit; these were set aside for later reflection on the findings rather than used as a framework for analysis. Critical discussions were held within and between sub-teams during this process, and the data relevant to each question were then synthesised.

### Assessment of risk of bias

Reviewers assessed and ranked source quality and relevance and provided rationales for their decisions drawing on the RAMESES Quality Standards for Realist Synthesis [31]. Key principles by which sources were assessed included: whether they contributed to the development or testing of programme theories; and, in the case of research sources, rigour: whether the studies used credible and trustworthy methods. We used an adapted checklist from the Critical Appraisal Skills Programme (CASP) to assess rigour of research-based sources. Additionally, an overall assessment rating of low, medium or high was assigned, relating to the source’s usefulness for the review, based on content with sufficient detail to inform the analysis, with ‘high’ rated sources prioritised during data synthesis.

### Data synthesis

We used a two-stage approach to synthesis; an initial analysis identified themes from the review data on what works for whom, how, and in what circumstances. A second, narrative synthesis, iteratively developed overarching themes through data interrogation and review team discussions. Statistical analysis based on any group or sub-group outcomes was outside the review scope and focus.

## Results

### Identification, screening and study selection

**Fig. 1:**
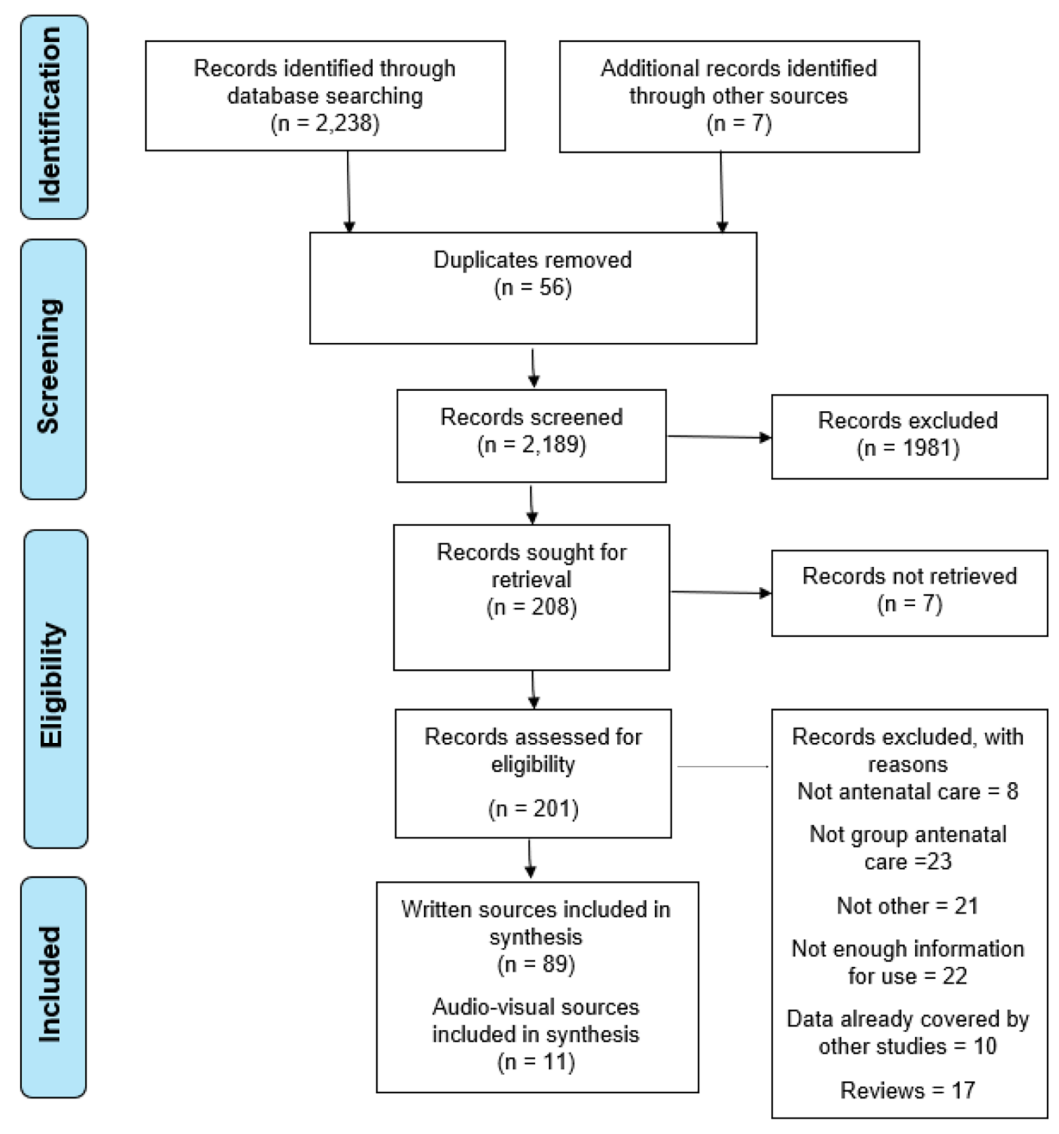
Consort diagram

The initial electronic search in 2015 produced 2,238 records with another seven sources obtained from hand searching of reference lists. After full screening, the review included 100 sources (of which 11 were audiovisual and the remaining 89 written sources) (see Fig. 1). An updated search in April 2019 identified 75 additional sources, of which 48 met the inclusion criteria and 27 were excluded following full text review (two of which were study team publications). Of those 48 sources, 15 were conference abstracts or posters which did not provide sufficient detail to add new insights to the analysis. Of the remaining 33 sources, there were no additional or divergent themes identified and they were therefore not included in this synthesis (summary details are given in Supplementary file 1 and 2). As a result, we concluded that analytical saturation in relation to theories of effect had been reached and no further search updates were conducted.

### Characteristics of included sources

Most sources were either cohort studies or opinion/expert reviews. Only four sources were from the Global South. Most sources (*n* = 77) related to populations and contexts in the United States. There were 14 studies or projects which had numerous sources reporting on outcomes or were commentaries, editorials or conference abstracts related to that study. The sources from the same project or study were linked for the review and data extraction to avoid data duplication.

The vast majority referred to a ‘standard’ CenteringPregnancy (*n* = 62). This follows the recommended schedule of ANC visits (lasting 90 min to two hours long) with women taking and recording their own health data, facilitative discussions, and activities to address important health topics, with private time with their provider. Of the CenteringPregnancy interventions, six described the model with ‘additions’ (including dental oral health components or specific topics relating to adolescents and youth). Another six sources described CenteringPregnancy with ‘adaptations’ where the private provider time was either scheduled outside of the main group session (i.e., either side of it), or where ANC visits involved a combination of one-to-one appointments and group sessions throughout pregnancy. There were seven sources describing non-CenteringPregnancy models of group ANC, which broadly described similar models of care to standard CenteringPregnancy.

### Findings

#### What works and for whom?

Evidence on the benefits for particular population groups was inconsistent when examining ‘what works’. We coded and categorised outcomes into four key domains: (1) experience (e.g., satisfaction), (2) clinical (e.g., mode of birth, birth weight), (3) health behaviours (i.e. smoking, breastfeeding), and (4) psychosocial (e.g. self-efficacy). We then coded population groups into 10 categories based on four population risk factors (social/demographic factors; medical, economic or none) within high income or low to middle income countries (see Table 1).

**Table 1:** Categories of population groups

	High income country	Low-income country
General	1. General population with no reported ‘risks’ 2. Unknown (not stated)	3. General population with no reported ‘risks’
Economic risk factors	4. Low income	-
Medical risk factors	5. High-risk 6. Low-risk	-
Social and/or demographic risk factors	7. Underserved 8. Military groups 9. Minority ethnic groups 10. Young	-

Mapping the outcome categories by population group categories generated no overall conclusive patterns as to what works for any particular population groups, although there were some indications of benefits for military families [33], for African American women [34] and for adolescent mothers [35].

Poor reporting and rationale for targeting particular population groups hampered comparisons. For example, some sources stated the targeted population groups were socially or medically high-risk but did not explain in detail or provide a clear rationale for why and how group ANC was expected to confer benefits. Others defined risk by the geography of an area such as where a clinic was located (i.e., low-income area) but did not explain this further in relation to the group care participants. Furthermore, being from a minority ethnic group was deemed high-risk due to the increased prevalence of poorer clinical outcomes at the population level, with little detailed understanding of how race and ethnicity were associated with poorer outcomes. For example, group ANC tended to have limited effectiveness compared with usual care in communities where women already had strong social support networks [36, 37]. Some sources also referred to the ‘Latina paradox’ whereby group ANC had little positive effect due to the already high levels of social support found within the Latin American population [37]. No studies examined the interconnected nature of multiple and compounding risk factors (i.e., through an intersectionality lens).

#### How (mechanisms of effect)?

Most sources drew on implicit rather than formal explicit theories to explain how group ANC might work to improve outcomes. Most sources also described CenteringPregnancy therefore they shared common theorised mechanisms of how group ANC might work. All implicit and explicit explanations were drawn out and coded, which generated six broad mechanisms of effect: (1) Social support; (2) Peer Learning; (3) Active Participation in Health; (4) Health Education; (5) Satisfaction with care; (6) Health Professional Development and Wellbeing (see Table 2).

**Table 2:** Theorised mechanisms of effect

Mechanism	Description	Example sources
1. Social support	Bringing women together in a group and receiving continuity of peers provides the opportunity for building supportive relationships and social capital. Furthermore, trust can form to share experiences and disclose concerns which can normalise pregnancy, encourage problem-solving, coping and resilience leading to reduced stress. This moves support to the community and reduces dependency on health services. Reference to theories of social capital and community development.	[38–51]
2. Peer learning	Learning occurs through peers who are deemed to share similar characteristics as themselves (in some cases socio-demographic but more often the pregnancy experience). Information and messages from peers are seen as more salient, relevant, and personalised therefore women are more likely to act on that knowledge. Highlights the value of different sources of knowledge and expertise and that peers can be positive role models. This modelling leads to greater confidence to take control of their own health by viewing others’ behaviours. Reference to social cognitive theory and theories of behaviour change.	[36, 43, 46, 47, 51–66]
3. Active participation in health	Learning occurs through active participation in health and doing things for oneself where self-checks, engaging in active discussions, and problem-solving places women at the centre of their own health. Shared health activities and engaging in women-led, group-based discussions supported more equal and trusting relationships between women and midwives.	[37, 39, 46, 62]
4. Health education	A group setting allows more time for ANC education and to cover a broader range and depth of a health curriculum. Group ANC is theorised as a space to deliver behavioural strategies through specialised content (e.g., dental care, HIV support) and practical demonstrations to increase the transaction of ‘expert’ knowledge and support for women to make appropriate choices for their health. Reference to behaviour change theories.	[41, 45, 55, 59, 67–70]
5. Satisfaction with care	A group setting enabled more time and continuity with a midwife and other healthcare professionals. Group ANC was seen as facilitating positive relationships between women and their healthcare provider, particularly where midwives are able to build relationships which are based on trust leading to greater satisfaction with care, better management of risks, and increased engagement with health services generally. Furthermore, groups allow better joined up care where other health professionals and invited speakers can attend groups to provide information (i.e., health visitors).	[33, 36, 50–53, 55, 59, 67, 71–74]
6. Health professional development and wellbeing	Midwives are able to provide richer and safer care with the increased time and continuity with women as well as through gaining the opportunity to develop their own knowledge with colleagues. This increases midwives job satisfaction which in turn translates to better care provided and reduced burn-out.	[45, 51, 75]

Most sources referred to a number of these mechanisms, but some focussed on one or two only; for example, transaction of knowledge and health persuasion messaging (an individualised theory) while others were instead focused on the exchange of peer knowledge and breaking down of traditional ‘expert knowledge’ sources (a collective theory of action).

Group ANC was believed to facilitate positive relationships between women and their healthcare provider where more time and continuity with midwives (and other health professionals) built additional trust leading to increased satisfaction and engagement with care, as well as management of risks that was more responsive [33, 36, 50–53, 55, 59, 67, 71–74]. Shared health activities and engaging in women-led, group-based discussions supported woman-midwife relationships that were more equal and trusting [37, 39, 46, 62]. Additionally, it was theorised that midwives deliver richer and safer care within group ANC models through more positive relationships with women and through gaining the opportunity to develop their own knowledge with colleagues [50, 75].

Sources referred to mechanisms relating specifically to the group element of care. For example, group modelling supported empowerment where women developed greater confidence to take control of their health by viewing others’ behaviours [46, 47, 53, 55, 59, 61–64, 66, 76]. It was theorised that group ANC provided peer and community support, allowing women to share and normalise experiences, whilst also gaining practical and relevant psychosocial support [38–44, 71]. Reduced stress and increased coping skills through pregnancy were anticipated [58, 77]. It was expected that peer support would reduce unnecessary reliance on health services and build more resilient communities [36, 41, 45–47, 50, 51].

An overarching mechanism was the reoccurring concept of empowerment as related to increasing women’s knowledge, ability to make better informed decisions (and behaviour changes) and building positive support networks with healthcare providers, own peers and their communities. However, most sources poorly theorised the concept of empowerment with different underpinning assumptions from individualistic and collective perspectives. For instance, nearly all theorised a link between greater knowledge and empowerment, whereby active learning approaches (e.g., peer-led group discussions) results in more relevant and salient knowledge, leading to empowerment in decision making and positive behaviour changes. This process of empowerment was largely conceptualised through an individualistic lens relating to self-efficacy and control over one’s health rather than broader concepts of empowerment which instead encompass collective and/or group levels of empowerment and/or paradigmatic shifts in care delivery. However, there was insufficient detail in the data to enable a fuller exploration of how such differences in the hypothesised underpinning mechanisms may influence the implementation, process or outcomes of group care implemented in different settings.

#### In what circumstances?

There were three main context factors related to implementation and delivery of group ANC models. Factors included: (1) Focus on the community and hyper-local level; (2) Shifting care out of hospitals, (3) Adapting to a different way of working.

##### Focus on the community and hyper-local level

Most group ANC models of care sought to focus on the community and hyper-local level (i.e., particular populations and areas of deprivation) to signify equal partnership between women and facilitators [78]. Group ANC models needed to be easily accessible at this level to recruit and reach women whilst also working at a scale to allow for an appropriate number to form a group size of 8–10. This posed a number of practical challenges for teams, mainly recruiting a desired number of women at similar gestations within local areas which required good targeting, scheduling and organisation [38, 71]. Focused recruitment strategies were needed to encourage women’s interest and engagement e.g., vouchers, automated reminders and involving local women in setting up and promoting groups [39, 79]. An opt-out recruitment (rather than opt-in) was also used with success in another study [38]. There was some evidence that a lack of childcare facilities discouraged multiparous women from attending group care [37, 79]. Engaging women in early pregnancy was considered important (particularly if any behaviour change was a desired outcome) [53, 67, 80], however engaging women too early could lead to high discontinuation rates for practical reasons or early pregnancy loss [52].

##### Shifting care outside hospitals

Most group ANC models were delivered in community settings rather than hospitals to aid accessibility, work at community and hyper-local level, and reach target populations. For example, an initiative targeting pregnant school pupils was held on a school site immediately after the end of the school day, which supported attendance [39, 79]. Sourcing suitable community venues, and the increased time taken to transport equipment and set up venues each week, was a recurring practical challenge [33, 45, 55, 71, 81]. Many community-based venues were often not immediately appropriate for medical tasks such as routine blood tests and accessing patient records [39, 75]. Group ANC required appropriate infrastructure and troubleshooting to manage the shift to care delivery outside of hospitals.

##### A different way of working

Groups were often set up and/or sustained by a small group of midwives or nurses who were committed to the concept, took ownership of the initiative and invested considerable time and effort to ensure its success [39]. The satisfaction gained from providing group ANC, working flexibly, making compromises where necessary and supporting each other enabled facilitators to sustain the model and overcome obstacles to implementation [38, 82]. Group ANC was usually a very new way of working for teams and services and there were key points of incompatibility to overcome. For example, group facilitation was not a well-developed skill for most midwives [83–85]. Adequate facilitator training was essential; and in its absence, both midwifery and medical professionals tended to adopt an overly didactic style, which was unsatisfying to women, leaving them feeling their concerns had not been addressed [52, 72, 86]. A didactic teaching style was deeply ingrained in some physicians [55], as reported in some Swedish and Canadian studies [52]. Women also needed to have a good understanding of the model otherwise there were confusions. For example, in one study, participants were not aware that group attendance replaced standard individual appointments and women tried to attend both [33]. Group ANC models also needed to consider the value this model offered within existing care. In one study, group ANC was less well received when set against a case-loading approach [45]. There was insufficient detail on national and local policies and health system factors to enable a full exploration of these broader context issues or to identify whether group care may have different impacts in settings with or without established and autonomous midwifery care, or in health systems which are universal or with a mix of private and subsidised care.

## Discussion

Overall, there was no clear pattern of ‘what works for whom, in what circumstances’. Although in some studies group care was targeted to more underserved groups or in more disadvantaged areas, and some studies have identified specific benefits for these groups [18] mechanisms such as peer learning and social support appeared salient for all groups. Variation in contexts, group ANC application or interpretation as well as which communities were involved may account for the inconsistency of findings. Our analysis did reveal some other important insights. We identified six interlinking mechanisms drawn out from mainly implicit descriptions. Mechanisms included: social support, peer learning, active participation in health, health education and satisfaction or engagement with care. A further theory related to impact on professional practice but was relatively under-developed. An overarching mechanism of empowerment featured across most studies, but most models largely adopted an individualistic lens despite the group/community focused approach. For example, some focused on the role of education, peers, and social support to change actions and behaviours. Others focused on broader paradigmatic shifts in professional-client relationships and the redistribution of power to women and communities. Conceptualisation of educational mechanisms drew on two somewhat different areas of pedagogical theory: one more focused on the emancipatory potential of the group approach to information and learning, whereas the other was rooted in a more transactional concept of education. The mechanisms of effect relating to empowerment were particularly important when considering which population group(s) to target for group ANC. There was poor reporting of populations, inadequate rationales for why particular populations were targeted and how the model was expected to confer benefits. For example, limited benefits were reported where women already had strong social support networks. No studies considered intersectionality of multiple and compounding risk factors. Few studies considered wider health system factors in shaping contexts, mechanisms and outcomes and most focused on site-specific context factors relating to implementation and individual/team level cultures. Much of the early conceptualisation and implementation of group ANC took place in the US, and it is possible that the model may function differently and have varying effects in different health systems, rather than simply in different local contexts or working with different populations and communities.

Our findings are largely in line with other research, including a previous Cochrane review demonstrating that there is insufficient evidence of benefits from this care model [16, 17] They are also concordant with a later scoping review to construct a conceptual framework for group well-child care [87] [where Gresh and colleagues identified guiding frameworks including adult and experiential learning theory social learning theory, Freire‘s pedagogy and several behaviour change theories]. We suggest there are inconsistencies in the evidence base due to variation in contexts, how the model is applied or interpreted as well as which communities are involved. For instance, our findings support other reviews which suggest that group ANC is likely to be most beneficial in groups and contexts with high levels of inequalities in access and experience of care, such as higher-risk or more vulnerable populations such as African American women and adolescents [18]. Another review focused on attributes that may support acceptability and effectiveness in LMICs and posited a generic model which was concordant with the mechanisms of effect we identified here, including empowerment and social support [21]. Since mechanisms of effect may have particular advantages in contexts where access and care inequalities exist, giving ‘women a voice for knowledge sharing and a sense of community support’ [21] may be of particular value. The concept of empowerment was present in a range of sources, but the meaning of empowerment was less often developed. Ongoing research by the review team suggests that empowerment may be mutually constructed through development of those facilitating as well as those participating in group care [21, 22], which is consonant with Freire’s emancipatory pedagogy as compared with a ’banking’ model of education [88].

### Strengths and weaknesses of the review

**Fig. 2:**
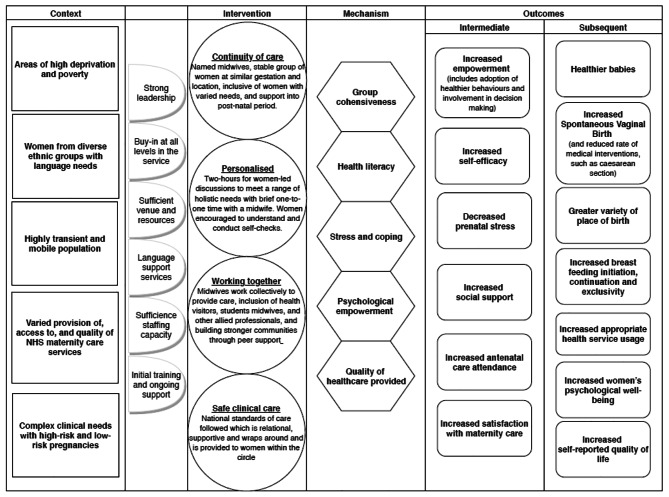
Pregnancy circles logic model

**Fig. 3:**
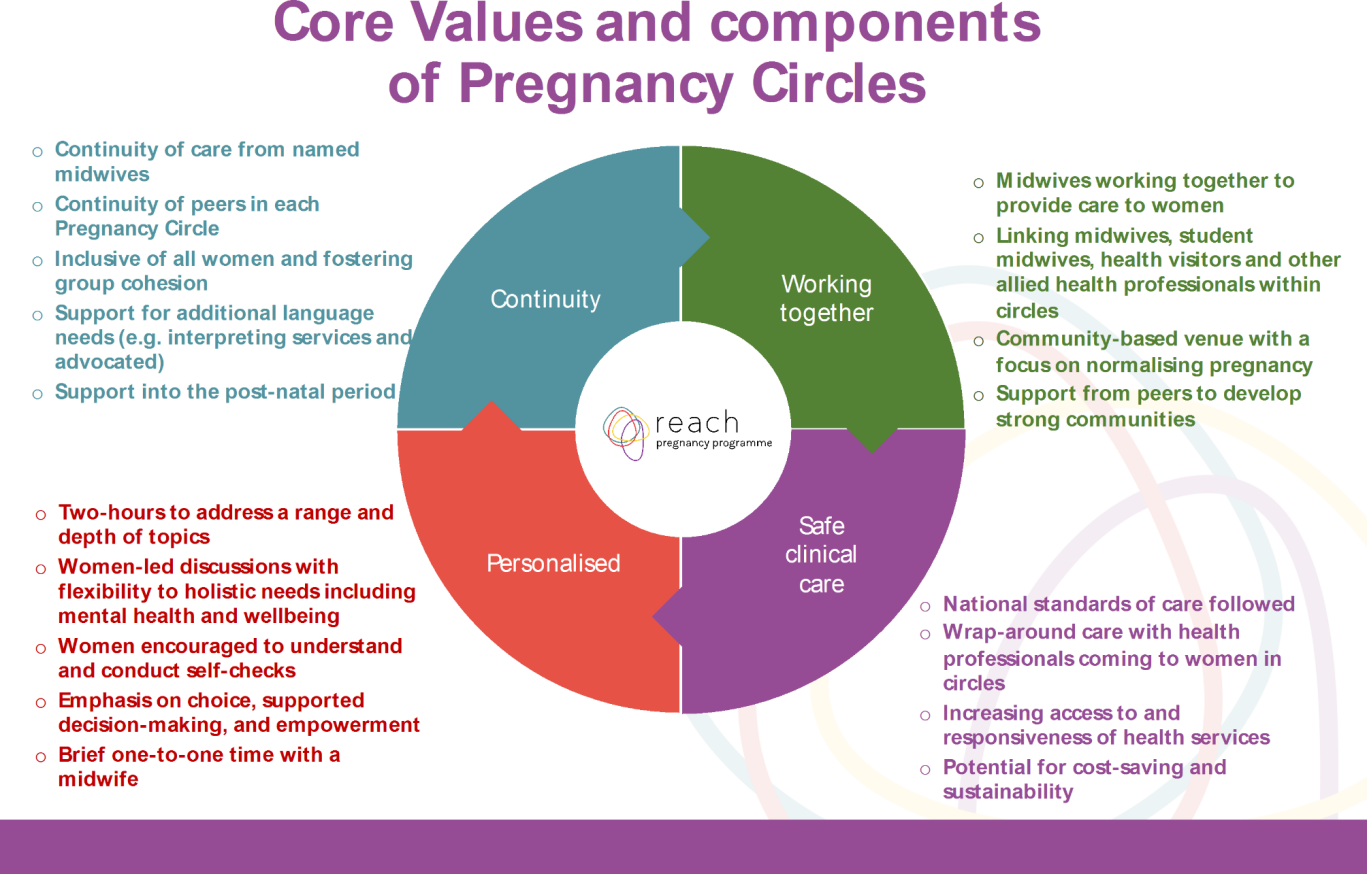
Pregnancy circles values and components

A systematic approach to searching for sources, including research and non-research literature helped to ensure a comprehensive set of sources, although inevitably some sources which may have been relevant will be missed by such a process. A realist approach helped to identify the potential mechanisms of effect for how group ANC ‘works’ with calls for more theoretical understanding about the concept of empowerment and how this relates to particular groups facing intersecting forms of inequality, disadvantage and discrimination. This approach also helped to provide more nuanced guidance on what to consider when implementing group ANC, including what features of the context are important. Our analysis however was limited by the lack of detail in study reporting, which meant some implicit understanding and insights had to be drawn out. Potential for researcher biases were handled through discussions and reviewing our own assumptions at review commencement to check how these may influence findings. In anticipation, each review team member drew a logic model at the outset, setting out their own theoretical propositions and assumptions about mechanisms of effect. For this reason, the team did not include their own developing work in the review, as we considered this may weight the analysis more heavily towards our own thinking. That said, the review work was intended to inform our thinking about possible mechanisms of effect of group care and took place over an extended period alongside our primary research, so it is likely that influences were mutual. Searches took place initially to feed into a feasibility study and development work for a bespoke model of group care to function in a UK NHS setting. A subsequent update identified no new themes relating to theories or mechanisms of effect. The team concluded that sufficient saturation was reached in the literature to inform future work to implement and evaluate this model of care (see Figs. 2 and 3). The review did not lead to any dissonant findings but instead provided a more developed consideration of possible mechanisms of effect for group care, as reflected in Fig. 2 and informed our Core Values and Components description for Pregnancy Circles (Fig. 3).

### Implications for researchers, care providers, and policymakers

This review of group ANC provides important implications for researchers, care providers and policymakers. Our review was hampered by the lack of study details particularly intervention and contextual descriptions and rationales for population group targeting. There is also a need for greater reporting quality and consistency. Future research would benefit from a clearer focus on mechanisms of effect, to ensure appropriate outcome measures are used, but also a clearer focus on who is expected to benefit and in which contexts. Further trials are essential, including detailed process evaluations exploring the role of care context and process, professional preparation and attitudes, the communities involved and how they experience group care. We identified few studies or other sources that examined the effects on care providers and could not identify theories of how any impact on professionals may have an impact on service users. We did not identify any negative impacts of more interactive and active participation of service users on professionals, although this is likely to be an important line for future research (for example, see [89]).

Much of the early conceptualisation and implementation of group ANC has taken place in the US, where the health financing system is not universal, access to healthcare is inequitable and midwives remain relatively marginal rather than mainstream healthcare providers. It is possible that the model may function differently and have varying effects in different health systems, rather than simply in different local contexts or working with different populations and communities. These should be considered in more depth in future studies and reviews. This review was undertaken alongside the conduct of a qualitative feasibility study and pilot trial. Both were intended to contribute to the conceptualisation and design of a contextually adapted model to be tested formally in a trial, with integral process evaluation. The findings of both studies were utilised to develop a logic and core values model, which helped to shape the trial intervention and provide a guide for the process evaluation, through clarifying how this model of care should be implemented in the study sites (see Figs. 2 and 3). An RCT with nested qualitative evaluation is currently in progress [28], following a successful pilot trial [90].

For providers and policymakers, we outline some key context factors which suggest a focus on supporting staff and teams to implement group ANC at a hyper-local community level and enabling the systems, infrastructure, time and training to shift care out of hospital settings and bring on broader paradigmatic shifts in care delivery and the women-provider relationship. Group ANC facilitators required support and learning to deliver the model in non-didactic ways and to bring out the benefits of the group dynamic. Further work is needed to examine the concept of empowerment, whether and how this may operate as an overarching mechanism of effect and in what circumstances.

## Electronic supplementary material

Below is the link to the electronic supplementary material.

Supplementary Material 1

## Data Availability

All data generated or analysed during this study are included in this published article (and its supplementary information files).
