# Supplementary material for: What are the mechanisms of effect of group antenatal care? A systematic realist review and synthesis of the literature
Source: BMC Pregnancy Childbirth. 2024 Oct 1;24:625. doi: 10.1186/s12884-024-06792-6 (PMC11446066; doi:10.1186/s12884-024-06792-6)
Supplement: Supplementary file 1 — Supplementary Material 1 [file 12884_2024_6792_MOESM1_ESM.docx]

# Appendices

**Appendix 1: Search terms**

1. ANC terms

Descriptor, keyword, subject: childbirth or Prenatal Care OR Prenatal Diagnosis OR Perinatal Care or Maternal Health Services OR Obstetrical Nursing or parent education or parent education program or mothers education or fathers education or Fathers [education] or Mothers [education]

Tiab: pre-natal or prenatal or peri-natal or perinatal or ante-natal or antenatal or childbirth or parturition or obstetr* or neonatal or neo-natal or midwife or midwives or matern* or antepartum or ante-partum or peripartum or peri-partum

AND

2. Group care terms

Subject: Group processes or group process

Tiab: care model* or model* of care or model* of antenatal care or model* of prenatal care or model* of ante-natal care or model* of pre-natal care or circle* adj2 (education or class or classes or screening* or assessment* or checkup* or check-up* or check up*) or Group education or group class* or group screening* or group assessment* or group checkup* or group check-up* or group check up* or Group Family Nurse Partnership* or gFNP

OR

Search 2

Tiab: CenteringPregnancy or Centering Pregnancy or (group antenatal or group prenatal or group ante-natal or group pre-natal) adj1 (care or education or class* or assessment* or checkup* or check-up* or check up*)

Date range: 1980

# Supplementary files

**Supplementary file 1: Summary table of sources included in the review**

| **Full citation** | **Source type** | **Design** | **Country of focus** | **Population group**  **of focus** | **Intervention** | **Usefulness rating** |
| --- | --- | --- | --- | --- | --- | --- |
| Allen J, Stapleton H, Tracy S, Kildea S. Is a randomised controlled trial of a maternity care intervention for pregnant adolescents possible? An Australian feasibility study. BMC medical research methodology. 2013 Dec;13(1):138. | Written  Empirical data | RCT (pilot) | Australia | Young  13 - 17 year olds | CenteringPregnancy | Low |
| Allen J, Kildea S, Stapleton H. How does group antenatal care function within a caseload midwifery model? A critical ethnographic analysis. Midwifery. 2015 May 1;31(5):489-97. | Written  Empirical data | Cohort | Australia | Young  19 - 22 years | CenteringPregnancy | High |
| Anderson C, Harris MS, Kovarik R, Skelton J. Discovering Expectant Mothers' Beliefs about Oral Health: An Application of the Centering Pregnancy Smiles® Program. International quarterly of community health education. 2009 Jul;30(2):115-40. | Written  Empirical data | Non-randomised trial | USA | Rural, isolated, few local services | CenteringPregnancySmiles [addition]  CenteringPregnancy with addition of oral health instructions and treatment from dentist and oral hygenist. | Medium |
| Andersson E, Christensson K, Hildingsson I. Parents' experiences and perceptions of group-based antenatal care in four clinics in Sweden. Midwifery. 2012 Aug 1;28(4):502-8. | Written  Empirical data | Qualitative | Sweden | General population group | CenteringPregnancy [adapted]  Physical examinations and check-ups provided in individual 10-min sessions after group session | High |
| Andersson E, Christensson K, Hildingsson I. Mothers’ satisfaction with group antenatal care versus individual antenatal care–a clinical trial. Sexual & Reproductive Healthcare. 2013 Oct 1;4(3):113-20. | Written  Empirical data | Non-randomised trial | Sweden | Not specified |  | High |
| Andersson E, Christensson K, Hildingsson I. Swedish Midwives' Perspectives of Antenatal Care Focusing on Group-Based Antenatal Care. International Journal of Childbirth. 2014 Oct 1;4(4):240. | Written  Empirical data | Qualitative | Sweden | General population group |  | High |
| Baldwin KA. Comparison of selected outcomes of CenteringPregnancy versus traditional prenatal care. Journal of Midwifery & Women's Health. 2006 Jul 1;51(4):266-72. | Written  Empirical data | Non-randomised trial | USA | Healthy pregnant women without medical/obstetric complications | CenteringPregnancy | Medium |
| Baldwin K, Phillips G. Voices along the journey: midwives’ perceptions of implementing the CenteringPregnancy model of prenatal care. The Journal of Perinatal Education. 2011;20(4):210. | Written  Empirical data | Qualitative | USA | Not specified |  | High |
| Barnes J, Henderson J. Summary of the formative evaluation of the first phase of the group-based family nurse partnership programme. Project Report. Department of Health, London, UK | Written  Empirical data | Cohort | UK | Under 25 years,  socially challenging | Group Family Nurse Partnership (gFNP)  30 meetings during pregnancy and into infancy (up to 12 months).  Women due within 4 - 6weeks, 2 hour meetings, self-checks, clinical care, information sharing, in a community venue. 1 midwife and 1 health visitor. | High |
| Barr WB, Aslam S, Levin M. Evaluation of a group prenatal care-based curriculum in a family medicine residency. Family Medicine-Kansas City. 2011 Nov 1;43(10):712. | Written  Empirical data | Cohort | USA | Not specified | CenteringPregnancy | Medium |
| Benediktsson I, McDonald SW, Vekved M, McNeil DA, Dolan SM, Tough SC. Comparing CenteringPregnancy® to standard prenatal care plus prenatal education. BMC pregnancy and childbirth. 2013 Jan;13(1):S5. | Written  Empirical data | Cohort | Canada | Region of high proportion of immigrants and low SES | CenteringPregnancy | High |
| Buzi RS, Smith PB. Project Passport: Engaging Pregnant Adolescents in a Journey of Self-Discovery and Commitment to the Future. Journal of Adolescent Health. 2013 Feb 1;52(2):S110. | Written  Empirical data | Cohort | USA | Adolescents  (15 - 18 years) | CenteringPregnancy [addition]  Addition of 'positive 13youth development exercises' into sessions | Medium |
| Smith PB, Buzi RS, Kozinetz CA, Peskin MF, Wiemann CM. Pregnant Adolescents' Family Formation and Perceived Partner Supportiveness in Early Pregnancy and Postpartum. Journal of Adolescent Health. 2015 Feb 1;56(2):S109. | Written  Empirical data | Non-randomised trial | USA | Young adolescents | CenteringPregnancy | Low |
| Carlson NS, Lowe NK. CenteringPregnancy: a new approach in prenatal care. MCN: The American Journal of Maternal/Child Nursing. 2006 Jul 1;31(4):218-23. | Written  Not empirical | Opinion/expert review | USA | n/a | CenteringPregnancy | Medium |
| Chesnut LW. Centering Pregnancy and Adverse Pregnancy Outcomes: An Evaluation of Group Prenatal Care in a Rural Western Kentucky Clinic (Doctoral dissertation, University of Alabama at Birmingham). | Written  Empirical data | Cohort | USA | General population group | CenteringPregnancySmiles [addition]  Addition of dental health component where all women receive an initial health exam by dentist with a treatment plan and two oral health education and demonstration sessions. | High |
| Chung LH, Gregorich SE, Armitage GC, Gonzalez‐Vargas J, Adams SH. Sociodemographic disparities and behavioral factors in clinical oral health status during pregnancy. Community Dentistry and Oral Epidemiology. 2014 Apr;42(2):151-9. | Written  Empirical data | Cohort | USA | Urban, Hispanic, well-educated | CenteringPregnancy Oral Health Promotion [addition]  Addition of dental examination and two oral health education sessions. | Medium |
| DeCesare JZ, Jackson JR. Centering Pregnancy: practical tips for your practice. Archives of Gynecology and Obstetrics. 2015 Mar 1;291(3):499-507. | Written  Not empirical | Opinion/expert review | USA | n/a | CenteringPregnancy | Medium |
| Devitt NF. Does the CenteringPregnancy Group Prenatal Care Program reduce preterm birth? The conclusions are premature. Birth. 2013 Mar;40(1):67-9. | Written  Not empirical | Opinion/expert review | USA | n/a | CenteringPregnancy | Medium |
| Doaee S, Nejati M, Heidari R, Haghollahi F. Women’s attitude to group prenatal care and their satisfaction. Journal of Pakistan Medical Association. 2013 Jan 1;63(1):50-4. | Written  Empirical data | Cohort | Iran | General population | n/a | Low |
| Gaestel A. (2013). Transforming prenatal care to lower infant mortality. Kaiser Health News, 6 August | Written  Not empirical | Opinion/expert review | USA | General population | CenteringPregnancy | Medium |
| Hale N, Picklesimer AH, Billings DL, Covington-Kolb S. The impact of Centering Pregnancy Group Prenatal Care on postpartum family planning. American journal of obstetrics and gynecology. 2014 Jan 1;210(1):50-e1. | Written  Empirical data | Cohort | USA | Medicaid | CenteringPregnancy | Medium |
| Hale N, Picklesimer A, Billings D, Covington-Kolb S. The effect of CenteringPregnancy Group prenatal care on enrollment in the post-partum family planning Medicaid waiver program. American Journal of Obstetrics & Gynecology. 2013 Jan 1;208(1):S55. | Written  Empirical data | Cohort | USA | Medicaid |  | Medium |
| Faucher MA. Group Prenatal Care may improve postpartum family planning service utilization. Journal of midwifery & women's health. 2014 Sep;59(5):538-9. | Written  Not empirical | Opinion/expert review | USA | Low income |  | High |
| Garretto D, Bernstein PS. CenteringPregnancy: an innovative approach to prenatal care delivery. American Journal of Obstetrics & Gynecology. 2014 Jan 1;210(1):14-5. | Written  Not empirical | Opinion/expert review | USA | Not specified |  | Medium |
| Fausett M, Gill B, Esplin M, Shields A, Staat B. 13: Centering Pregnancy is associated with fewer early, but not overall, preterm deliveries. American Journal of Obstetrics & Gynecology. 2014 Jan 1;210(1):S9. | Written  Empirical data | RCT | USA | Military | CenteringPregnancy | High |
| Foster GA, Alviar A, Neumeier R, Wootten A. A Tri‐Service Perspective on the Implementation of a Centering Pregnancy Model in the Military. Journal of Obstetric, Gynecologic, & Neonatal Nursing. 2012 Mar;41(2):315-21. | Written  Not empirical | Opinion/expert review | USA | Military women | CenteringPregnancy | High |
| Gaudion A, Menka Y, Demilew J, Walton C, Yiannouzis K, Robbins J, Rising SS, Bick D. Findings from a UK feasibility study of the CenteringPregnancy® model. British Journal of Midwifery. 2011 Dec;19(12):796-802. | Written  Empirical data | Cohort | UK | General population, excluding non-English speakers | CenteringPregnancy | High |
| Gogel L, Zielinski R, Deibel M, Kothari C. Improving Maternal and Infant Health through CenteringPregnancy: Results of a 2‐Year Retrospective Chart Review Using a Matched Comparison Design. Journal of midwifery & women's health. 2013 Sep;58(5):584-5. | Written  Empirical data | Non-randomised trial | USA | Not specified | CenteringPregnancy | Medium |
| Gonzales-Pacheco D, Cox K, Murray-Krezan C. Gestational weight gain in women receiving prenatal care in rural midwifery clinics (LB313). The FASEB Journal. 2014 Apr;28(1_supplement):LB313. | Written  Empirical data | Cohort | USA | Not specified | Group Prenatal Care  No detail provided | Low |
| Grady MA, Bloom KC. Pregnancy outcomes of adolescents enrolled in a CenteringPregnancy program. Journal of midwifery & women's health. 2004 Sep 1;49(5):412-20. | Written  Empirical data | Cohort | USA | Adolescents (age 15-19 years | CenteringPregnancy [addition]  Some additions to include adolescent issues including STIs, abuse, parenting experiences. | Medium |
| Griswold, C.H., Nasso, J.T., Swider, S., Ellison, B.R., Griswold, D.L. and Brooks, M., 2013. The prenatal care at school program. The Journal of School Nursing, 29(3), pp.196-203. | Written  Empirical data | Cohort | USA | Adolescents (12 - 21 years, mean 15) | Prenatal Care at School (PAS)  8 sessions held in school setting after school, involving routine prenatal assessment and education. | High |
| Thielen K. Exploring the group prenatal care model: A critical review of the literature. The Journal of perinatal education. 2012 Jan 1;21(4):209-18. | Written  Not empirical | Opinion/expert review | USA | n/a | CenteringPregnancy | Medium |
| Heberlein E. The comparative effectiveness of group prenatal care on women's psychosocial health. PhD thesis. 2014 | Written  Empirical data | Cohort | USA | Underserved - social risk | CenteringPregnancy | High |
| Heberlein E, Picklesimer A, Covington-Kolb S, Boggess K, Moss K. Centering pregnancy group prenatal care does not change biomarker or self-reported stress measures. American Journal of Obstetrics & Gynecology. 2015 Jan 1;212(1):S164-5. | Written  Empirical data | Cohort | USA | Underserved - social risk |  | High |
| Ickovics JR, Kershaw TS, Westdahl C, Rising SS, Klima C, Reynolds H, Magriples U. Group prenatal care and preterm birth weight: results from a matched cohort study at public clinics. Obstetrics & Gynecology. 2003 Nov 1;102(5):1051-7. | Written  Empirical data | Cohort | USA | Medicaid | CenteringPregnancy (standard) | High |
| Ickovics JR, Kershaw TS, Westdahl C, Magriples U, Massey Z, Reynolds H, Rising SS. Group prenatal care and perinatal outcomes: a randomized controlled trial. Obstetrics and gynecology. 2007 Aug;110(2 Pt 1):330. | Written  Empirical data | RCT | USA | Medicaid | CenteringPregnancy | High |
| Ickovics JR, Reed E, Magriples U, Westdahl C, Schindler Rising S, Kershaw TS. Effects of group prenatal care on psychosocial risk in pregnancy: results from a randomised controlled trial. Psychology and Health. 2011 Feb 1;26(2):235-50. | Written  Empirical data | RCT | USA | Medicaid |  | High |
| Romano AM. Research summaries for normal birth. The Journal of perinatal education. 2007;16(3):53. | Written  Not empirical | Opinion/expert review | USA | n/a |  | Medium |
| Nielsen PE. Group Prenatal Care and Perinatal Outcomes: A Randomized Controlled Trial. Obstetrics & Gynecology. 2008 Apr 1;111(4):993. | Written  Not empirical | Opinion/expert review | USA | n/a |  | High |
| Jafari F, Eftekhar H and Fotouhi A ; Mohammad K ; Hantoushzadeh S ;. (2010). Comparison of maternal and neonatal outcomes of group versus individual prenatal care: a new experience in Iran. Health Care for Women International, 31(7), pp.571-584. | Written  Empirical data | RCT | Iran | Not specified | Group antenatal care model (not named)  8-10 women, 10 sessions during pregnancy, 90-120mins per session. Group discussions focusing on education and skills-building, promotion of self-care activities, non-didactic, prenatal care in group setting. | High |
| Jafari F, Eftekhar H, Mohammad K, Fotouhi A. Does group prenatal care affect satisfaction and prenatal care utilization in Iranian pregnant women?. Iranian Journal of Public Health. 2010;39(2):52. | Written  Empirical data | RCT | Iran | Not specified |  | High |
| Kennedy HP, Farrell T, Paden R, Hill S, Jolivet R, Willetts J, Rising SS. “I wasn't alone”—a study of group prenatal care in the military. The Journal of Midwifery & Women’s Health. 2009 May 6;54(3):176-83. | Written  Empirical data | Qualitative | USA | Military women | CenteringPregnancy | Medium |
| Kennedy HP, Farrell T, Paden R, Hill S, Jolivet RR, Cooper BA, Schindler Rising S. A randomized clinical trial of group prenatal care in two military settings. Military medicine. 2011 Oct 1;176(10):1169-77. | Written  Empirical data | RCT | USA | Military women |  | Medium |
| Kennedy HP, Braun LA, Farrell T, Hill S, Jolivet R, Paden R, Rising SS, Tramantana J. Power, Energy, & Bonds: Military Providers' Perceptions of Group Prenatal Care. Nursing Research. 2013. 62 (2): E32-E32 | Written  Empirical data | Qualitative | USA | Military women |  | Low |
| Kershaw TS, Magriples U, Westdahl C, Rising SS, Ickovics J. Pregnancy as a window of opportunity for HIV prevention: effects of an HIV intervention delivered within prenatal care. American Journal of Public Health. 2009 Nov;99(11):2079-86. | Written  Empirical data | RCT | USA | Young women (14 - 25 years) | CenteringPregnancyPlus [addition]  Addition of HIV prevention education | High |
| Klima CS. Centering pregnancy: a model for pregnant adolescents. The Journal of Midwifery & Women’s Health. 2003 May 6;48(3):220-5. | Written  Not empirical | Opinion/expert review | USA | Adolescents | CenteringPregnancy | Medium |
| Klima C, Norr K and Vonderheid S ; Handler A ;. (2009). Introduction of CenteringPregnancy in a Public Health Clinic. Journal of Midwifery and Women's Health, 54(1), pp.27-34. | Written  Empirical data | Qualitative study | USA | Low-income African-American women. | CenteringPregnancy | Medium |
| Kolb KH, Picklesimer AH, Covington-Kolb S, Hines L. Centering pregnancy electives: a case study in the shift toward student-centered learning in medical education. Journal of the South Carolina Medical Association (1975). 2012 Aug;108(4):103-5. | Written  Not empirical | Opinion/expert review | USA | Medical students as potential/future facilitators | CenteringPregnancy | Low |
| Lathrop B, Pritham UA. A pilot study of prenatal care visits blended group and individual for women with low income. Nursing for women's health. 2014 Dec 1;18(6):462-74. | Written  Empirical data | Cohort | USA | Underserved, uninsured | Healthy Pregnancy, Healthy childbirth, Healthy Parenting (HPCP)  Elements of CenteringPregnancy while maintaining individual visits where one group session is provided at each semester with individual checks at the end of the session. No inclusion of self-checks and different women in each session. Other appointments during pregnancy are 1:1. | Medium |
| Law AJ, Kennedy HP. Group prenatal care with an urban Latina population: An interpretive field study. Journal of Midwifery and Womens Health. 2007 52 (5): 533-533) | Written  Empirical data | Cohort | USA | Military | CenteringPregnancy | Low |
| Little SH, Motohara S, Miyazaki K, Arato N, Fetters MD. Prenatal group visit program for a population with limited English proficiency. The Journal of the American Board of Family Medicine. 2013 Nov 1;26(6):728-37. | Written  Empirical data | Cohort | USA | Japanese women in USA with limited English proficiency | CenteringPregnancy [adapted] 5 group sessions to allow for other appointments to be private (1:1). Faciltiated by family physician and a registered nurse. Partners attended. | Medium |
| Maier B J. (2013). Antenatal group care in a Midwifery Group Practice - A midwife' perspective. Women and Birth, 26(1), pp.87-89. | Written  Not empirical | Opinion/expert review | Australia | General | CenteringPregnancy |  |
| Massey Z, Rising SS, Ickovics J. CenteringPregnancy group prenatal care: promoting relationship‐centered care. Journal of Obstetric, Gynecologic & Neonatal Nursing. 2006 Mar 1;35(2):286-94. | Written  Not empirical | Opinion/expert review | USA | General | CenteringPregnancy | Medium |
| McDonald SD, Sword W, Eryuzlu LE, Biringer AB. A qualitative descriptive study of the group prenatal care experience: perceptions of women with low-risk pregnancies and their midwives. BMC pregnancy and childbirth. 2014 Dec;14(1):334. | Written  Empirical data | Qualitative | Canada | Medical low risk | CenteringPregnancy [adapted] - referred to as 'Connecting Pregnancy'  Derivation of CenteringPregnancy where women recieve 1:1 appointments until mid-pregnancy, then nine, two-hour sessions monthly until 28 weeks then biweekly. Up to 10 women of similar gestation, women encouraged to self-check, 5 min 1:1 with midwife, discussions and videos. | High |
| McLean K. Pregnancy-related depression and maternal-child health: A comparison of prenatal care formats. Wheaton College; 2013. | Written  Empirical data | Non-randomised trial | USA | African American and Hispanic women in medically underserved areas | CenteringPregnancy | High |
| McNeil DA, Vekved M, Dolan SM, Siever J, Horn S, Tough SC. A qualitative study of the experience of CenteringPregnancy group prenatal care for physicians. BMC pregnancy and childbirth. 2013 Jan;13(1):S6. | Written  Empirical data | Qualitative | Canada | General | CenteringPregnancy | Medium |
| Moos MK. Prenatal care: limitations and opportunities. Journal of Obstetric, Gynecologic & Neonatal Nursing. 2006 Mar 1;35(2):278-85. | Written  Not empirical | Opinion/expert review | USA | Not specified | CenteringPregnancy | Medium |
| Novick G, Sadler LS, Knafl KA, Kennedy HP, Groce NE. Implementing Group Prenatal Care In 2 Urban Clinics. The Journal of Midwifery & Women’s Health. 2011 Sep;56(5):527-8. | Written  Empirical data | Qualitative | USA | Predominantly African American or Hispanic | CenteringPregnancy | Medium |
| Novick G. CenteringPregnancy and the current state of prenatal care. Journal of Midwifery & Women's Health. 2004 Sep 1;49(5):405-11. | Written  Not empirical | Opinion/expert review | USA | n/a |  | Low |
| Novick G, Sadler LS, Knafl KA, Groce NE, Kennedy HP. The intersection of everyday life and group prenatal care for women in two urban clinics. Journal of health care for the poor and underserved. 2012 May;23(2):589. | Written  Empirical data | Qualitative | USA | Low income |  | High |
| Novick G, Sadler L S and Kennedy H P; Cohen S S; Groce N E; Knafl K A;. (2011). Women's experience of group prenatal care. Qualitative health research, 21(1), pp.97-116. | Written  Empirical data | Qualitative | USA | Low income, African American or Hispanic |  | High |
| Novick G, Reid AE, Lewis J, Kershaw TS, Rising SS, Ickovics JR. Group prenatal care: model fidelity and outcomes. Am J Obstet Gynecol. 2013;209:112.e1–6. | Written  Empirical data | Quantitative | USA | Low income, African American or Hispanic |  |  |
| Novick G, Sadler LS, Knafl KA, Groce NE, Kennedy HP. In a hard spot: providing group prenatal care in two urban clinics. Midwifery. 2013 Jun 1;29(6):690-7. | Written  Empirical data | Qualitative | USA | Low income, African American or Hispanic |  | High |
| O’Neill M, Macones GA. Ambulatory Obstetric Care. Clinical obstetrics and gynecology. 2012 Sep 1;55(3):714-21. | Written  Not empirical | Opinion/expert review | USA | General population | CenteringPregnancy [adapted]  1:1 time first followed by group discussions. | Low |
| Patil CL, Abrams ET, Klima C, Kaponda CP, Leshabari SC, Vonderheid SC, Kamanga M, Norr KF. CenteringPregnancy-Africa: a pilot of group antenatal care to address Millennium Development Goals. Midwifery. 2013 Oct 1;29(10):1190-8. | Written  Empirical data | Cohort | Malawi/Tanzania | General population | CenteringPregnancy | High |
| Phillippi JC, Myers CR. " I Don't Want to Put Everything Out There"-A Qualitative Study of Reasons Appalachian Women Decline Centering Pregnancy. Journal of Midwifery and Womens Health. 2012. 57 (5): 538-538. | Written  Empirical data | Qualitative | USA | Low income, rural | CenteringPregnancy | Medium |
| Phillippi JC, Myers CR. Reasons women in Appalachia decline CenteringPregnancy care. Journal of midwifery & women's health. 2013 Sep 1;58(5):516-22. | Written  Empirical data | Qualitative | USA | Low income, rural |  | High |
| Picklesimer AH, Billings D, Hale N, Blackhurst D, Covington-Kolb S. The effect of CenteringPregnancy group prenatal care on preterm birth in a low-income population. American Journal of Obstetrics and Gynecology. 2012 May 1;206(5):415-e1. | Written  Empirical data | Cohort | USA | Low income | CenteringPregnancy | High |
| Picklesimer AH, Billings D, Hale N, Blackhurst D, Covington-Kolb S. The effect of CenteringPregnancy group prenatal care on preterm birth in a low-income population [editorial]. Obstretrical and Gynecological Survey. 2012, 206(5):415-e1. | Written  Empirical data | Cohort | USA | Low risk women |  | Medium |
| Quinn MT, Murtha AP, MacDonald AG. Pregnancy outcomes in centering pregnancy compared with traditional prenatal care. Obstretric and Gynecology. 2008. 111(4): 51S-51S | Written  Empirical data | Cohort | USA | General | CenteringPregnancy | Low |
| Raymond JE, Foureur MJ, Davis DL. Gestational weight change in women attending a group antenatal program aimed at addressing obesity in pregnancy in New South Wales, Australia. Journal of midwifery & women's health. 2014 Jul;59(4):398-404. | Written  Empirical data | Cohort | Australia | Obese (BMI>30) | Group antenatal care model (not named)  8-sessions (7 antenatal and 1 postnatal), 2-hours, 2 midwives providing continuity of carer. Inclusion of dietician, physiotherapist. No more than 12 women of similar gestation, community setting. | Medium |
| Anon 2011. Group approach to prenatal care garners enthusiasm among patients and providers. Disease management advisor, 7(9), pp.137-141 | Opinion | n/a | USA | Not specified | Centering | Medium |
| Rising, S.S. 1998  Centering pregnancy. An interdisciplinary model of empowerment. Journal of nurse-midwifery, 43(1), pp.46-54. | Opinion | n/a | n/a | n/a | Centering | High |
| Rising SS, Kennedy H P and Klima C S;. (2004). Redesigning prenatal care through CenteringPregnancy. Journal of Midwifery and Women's Health, 49(5), pp.398-404. | Opinion | n/a | n/a | n/a | Centering | High |
| Risisky D, Asghar SM, Chaffee M, DeGennaro N. Women’s perceptions using the CenteringPregnancy model of group prenatal care. The Journal of perinatal education. 2013;22(3):136. | Written  Empirical data | Qualitative | USA | General population group | CenteringPregnancy | Medium |
| Robertson B, Aycock DM, Darnell LA. Comparison of centering pregnancy to traditional care in Hispanic mothers. Maternal and child health journal. 2009 May 1;13(3):407. | Written  Empirical data | Non-randomised trial | USA | Hispanic | CenteringPregnancy | Low |
| Schellinger M, Abernathy M, Foxlow L, Carter A, Bastawros D, Haas D. Improved outcomes for Hispanic patients with gestational diabetes using the Centering Pregnancy group prenatal care model. American Journal of Obstetrics & Gynecology. 2013 Jan 1;208(1):S128. | Written  Empirical data | Cohort | USA | Women with diabetes | CenteringPregnancy | Low |
| <https://www.youtube.com/watch?v=SEFA4uL3-H4> | Audiovisual Not empirical | Opinion/expert review | USA | Not specified | CenteringPregnancy | Low |
| <https://www.youtube.com/watch?v=JPd12Vl2NgE> | Audiovisual Not empirical | Opinion/expert review | USA | Not specified | CenteringPregnancy | Low |
| <https://www.youtube.com/watch?v=WSGqs_XcWXk> | Audiovisual Not empirical | Opinion/expert review | USA |  | CenteringPregnancy | Low |
| <https://www.youtube.com/watch?v=jUQCx-ptg7k> | Audiovisual Not empirical | Opinion/expert review | USA |  | CenteringPregnancy [adapted]   Adapted where self-care activities and 1:1 at same time at the start followed by group discussions | Medium |
| <https://www.youtube.com/watch?v=KGwglKB80es> | Audiovisual Not empirical | Opinion/expert review | USA | Not specified | CenteringPregnancy | Low |
| <https://www.youtube.com/watch?v=_UVVTEVwch4> | Audiovisual Not empirical | Opinion/expert review | USA |  | CenteringPregnancy | Low |
| <https://www.youtube.com/watch?v=iqvAOy3zK9Q> | Audiovisual Not empirical | Opinion/expert review | USA | Not specified | CenteringPregnancy | Low |
| <https://www.youtube.com/watch?v=NNEPg0ok8jw> | Audiovisual Not empirical | Opinion/expert review | USA | Not specified | CenteringPregnancy | Low |
| <https://www.youtube.com/watch?v=KX8L8ZsHunk> | Audiovisual Not empirical | Opinion/expert review | USA | Spanish-speaking women | CenteringPregnancy | Medium |
| <https://www.youtube.com/watch?v=3Mf3Oe45Uuk> | Audiovisual Not empirical | Opinion/expert review | USA | Latina women | CenteringPregnancy | Medium |
| <https://www.youtube.com/watch?v=kUSVFsrQbTs> | Audiovisual Not empirical | Opinion/expert review | USA |  | CenteringPregnancy | Medium |
| Shakespear K, Waite PJ, Gast J. A comparison of health behaviors of women in centering pregnancy and traditional prenatal care. Maternal and child health journal. 2010 Mar 1;14(2):202-8. | Written  Empirical data | Cohort | USA | General population group | CenteringPregnancy [adapted]  Adapted where self-checks and 1:1 happen in first 30 mins following by 90mins of group education | High |
| Tandon SD, Cluxton-Keller F, Colon L, Vega P, Alonso A. Improved adequacy of prenatal care and healthcare utilization among low-income Latinas receiving group prenatal care. Journal of Women's Health. 2013 Dec 1;22(12):1056-61. | Written  Empirical data | Non-randomised trial | USA | Low income | CenteringPregnancy | Low |
| Tandon S D, Colon L and Vega P ; Murphy J ; Alonso A ;. (2012). Birth Outcomes Associated with Receipt of Group Prenatal Care Among Low-Income Hispanic |  |  |  |  |  |  |
| Tanner‐Smith EE, Steinka‐Fry KT, Lipsey MW. Effects of CenteringPregnancy group prenatal care on breastfeeding outcomes. Journal of midwifery & women's health. 2013 Jul;58(4):389-95. | Written  Empirical data | Non-randomised trial | USA | Minority groups | CenteringPregnancy | Medium |
| Tanner-Smith EE, Steinka-Fry KT, Lipsey MW. The effects of CenteringPregnancy group prenatal care on gestational age, birth weight, and fetal demise. Maternal and child health journal. 2014 May 1;18(4):801-9. | Written  Empirical data | Non-randomised trial | USA | African Americans, not high clinical risk |  | Medium |
| Tanner-Smith EE, Steinka-Fry KT, Gesell SB. Comparative effectiveness of group and individual prenatal care on gestational weight gain. Maternal and child health journal. 2014 Sep 1;18(7):1711-20. | Written  Empirical data | Non-randomised trial | USA | Mainly minority groups |  | Medium |
| Teate A, Leap N, Rising SS, Homer CS. Women's experiences of group antenatal care in Australia—the CenteringPregnancy Pilot Study. Midwifery. 2011 Apr 1;27(2):138-45. | Written  Empirical data | Cohort | Australia | Low risk women | CenteringPregnancy | High |
| Teate A, Leap N, Homer CS. Midwives’ experiences of becoming CenteringPregnancy facilitators: A pilot study in Sydney, Australia. Women and Birth. 2013 Mar 1;26(1):e31-6. | Written  Empirical data | Qualitative | Australia | Not specified |  | High |
| Trudnak TE, Arboleda E, Kirby RS, Perrin K. Outcomes of Latina women in CenteringPregnancy group prenatal care compared with individual prenatal care. Journal of midwifery & women's health. 2013 Jul 1;58(4):396-403. | Written  Empirical data | Cohort | USA | Latina, Spanish-speaking women | CenteringPregnancy | High |
| Vonderheid SC, Carrie SK, Norr KF, Grady MA, Westdahl CM. Using focus groups and social marketing to strengthen promotion of group prenatal care. Advances in Nursing Science. 2013 Oct 1;36(4):320-35. | Written  Empirical data | Qualitative | USA | n/a | CenteringPregnancy | High |
| Wedin K, Molin J, Svalenius EL. Group antenatal care: new pedagogic method for antenatal care—a pilot study. Midwifery. 2010 Aug 1;26(4):389-93. | Written  Empirical data | Non-randomised trial | Sweden | General population group | Group antenatal care model (not named)  Parent education with medical check-ups. Groups of around 6 women. 1 hour of group discussions with second hour of women with 10min 1:1 checks while discussions continue. All women still get 1:1 appointments - group is in addition. | Medium |
| Xaverius PK, Grady MA. Centering pregnancy in Missouri: a system level analysis. The Scientific World Journal. 2014;2014. | Written  Empirical data | Cohort | USA | Not specified | CenteringPregnancy | Medium |

**Supplementary file 2: Summary table of sources included in the review update**

| **Full citation** | **Country** | **Outline of study and whether any new data relevant to the review – that would amend or enhance the analysis** |
| --- | --- | --- |
| Abrams, J. A., Forte, J., Bettler, C., & Maxwell, M. (2018). Considerations for implementing group‐level prenatal health interventions in low‐resource communities: lessons learned from Haiti. Journal of Midwifery & Women's Health, 63(1), 121-126. | Haiti | No detailed description of the model and how it is intended to work beyond a brief reference to findings of other studies. The main focus is how to implement this in a resource-constrained context. Challenges noted include language, literacy, space, cultural appropriateness of intervention content, and socio-political climate. |
| Adams, C., & Thomas, S. P. (2018). Alternative prenatal care interventions to alleviate Black–White maternal/infant health disparities. Sociology compass, 12(1), e12549. | USA | Discusses the maternal health literature that critiques standard prenatal care in the United States by drawing on intersectionality, medicalization, and fundamental causation theories. It refers to existing theories in relation to group care, including empowerment, social support and the learning from peers. The theories cited are those already identified in the review but this article’s focus highlights the salience of these for black women in the US and posits that group approaches may have a particular appeal for them. |
| Brumley, J., Cain, M. A., Stern, M., & Louis, J. M. (2016). Gestational weight gain and breastfeeding outcomes in group prenatal care. Journal of Midwifery & Women's Health, 61(5), 557-562. | USA | A small case control study to compare gestational weight gain in women choosing and attending group prenatal care compared with standard individual prenatal care. It refers to existing theories already identified in the review including empowerment, through encouraging engagement and participation in care, and selfcare which may encourage healthy behaviours. No differences in weight gain were found but choosing group care was associated with a higher exclusive breastfeeding rate. |
| Carter, E. B., Barbier, K., Sarabia, R., Macones, G. A., Cahill, A. G., & Tuuli, M. G. (2017). Group versus traditional prenatal care in low-risk women delivering at term: a retrospective cohort study. Journal of Perinatology, 37(7), 769-771. | USA | Retrospective cohort study with matched controls of women with singleton, term pregnancies who participated in group care compared with traditional care. Group care was associated with a lower risk of low birth weight, of CS birth, low Apgar score or admission to higher-level neonatal care.  Article included no discussion of theories relating to the hypothesised benefits, although these were covered in a 2016 systematic review by the same team. |
| Chae, S. Y., Chae, M. H., Kandula, S., & Winter, R. O. (2017). Promoting improved social support and quality of life with the CenteringPregnancy® group model of prenatal care. Archives of women's mental health, 20, 209-220. | USA | Prospective cohort study to assess impact of Centering on perceived social support and quality of life, finding improved scores in both. Authors refer to existing theories that underpin the hypothesised effect including social support, self-efficacy, empowerment and health education and point to the theories of social support as a stress-buffer that may reduce distress and enhance quality of life. |
| Chen, L., Crockett, A. H., Covington-Kolb, S., Heberlein, E., Zhang, L., & Sun, X. (2017). Centering and Racial Disparities (CRADLE study): rationale and design of a randomized controlled trial of centeringpregnancy and birth outcomes. BMC pregnancy and childbirth, 17, 1-13. | USA | Protocol for a trial of Centering pregnancy to examine impact on preterm birth. The model is hypothesised to reduce PTB and racial disparities through increased patient-provider interaction time, developing trust and understanding and empowering and promoting self-care and positive behavioural changes. |
| Cunningham SD, Lewis JB, Thomas JL, Grilo SA, Ickovics JR. Expect With Me: development and evaluation design for an innovative model of group prenatal care to improve perinatal outcomes. BMC pregnancy and childbirth. 2017 Dec;17:1-3. | USA | Account of a Group care approach with a novel integrated IT platform (which appears to be an electronic patient-held record). Authors refer to existing theories of benefit as: improved learning and skills development, attitude change and motivation, enhanced insight through sharing of common experiences and social support and facilitating development of new community norms for health-enhancing behaviours. They posit that the group approach enhances health behaviours and decision making, and helps to connect providers and patients. |
| Cunningham, S. D., Lewis, J. B., Shebl, F. M., Boyd, L. M., Robinson, M. A., Grilo, S. A., ... & Ickovics, J. R. (2019). Group prenatal care reduces risk of preterm birth and low birth weight: a matched cohort study. Journal of women's health, 28(1), 17-22. | USA | Retrospective matched cohort study examined the impact of group prenatal care on preterm birth and low birth weight in a large metropolitan hospital, accounting for patient adherence, over an 8.5-year period.  Focused on outcomes only with no additional data on theories of effect. |
| DeCesare JZ, Hannah D, Amin R. Postpartum Contraception Use Rates of Patients Participating in the Centering Pregnancy Model of Care Versus Traditional Obstetrical Care. The Journal of reproductive medicine. 2017 Jan 1;62(1-2):45-9. | USA | Retrospective chart review focused on postpartum contraceptive rates  Focused on outcomes only.  No relevant data included. |
| Earnshaw VA, Rosenthal L, Cunningham SD, Kershaw T, Lewis J, Rising SS, Stasko E, Tobin J, Ickovics JR. Exploring group composition among young, urban women of color in prenatal care: Implications for satisfaction, engagement, and group attendance. Women's Health Issues. 2016 Jan 1;26(1):110-5. | USA | Analyses explored composition of groups in terms of age, race, ethnicity, and language, suggesting diverse age composition seems to be associated with young women's engagement in care, including young women of colour.  No new theories but useful exploration regarding group composition. |
| Eluwa GI, Adebajo SB, Torpey K, Shittu O, Abdu-Aguye S, Pearlman D, Bawa U, Olorukooba A, Khamofu H, Chiegli R. The effects of centering pregnancy on maternal and fetal outcomes in northern Nigeria; a prospective cohort analysis. BMC Pregnancy and Childbirth. 2018 Dec;18(1):1-0. | Nigeria | Prospective cohort study assessed the effect of Centering pregnancy groups on the uptake of antenatal care, facility delivery and immunization rates for infants.  No relevant theory data included. |
| Felder JN, Epel E, Lewis JB, Cunningham SD, Tobin JN, Rising SS, Thomas M, Ickovics JR. Depressive symptoms and gestational length among pregnant adolescents: Cluster randomized control trial of CenteringPregnancy® plus group prenatal care. Journal of consulting and clinical psychology. 2017 Jun;85(6):574. | USA | Cluster randomised trial focused on whether this model can reduce depressive symptoms in adolescents, given the clinical benefits found for adolescent mothers in some studies. Refers to existing evidence that group care provides opportunities for social support, and that participants learn stress reduction techniques and communication skills, which may improve psychosocial functioning. Significant reductions in depressive symptoms were observed and these may be associated with rates of preterm birth and gestational age at birth. |
| Fuentes-Rivera E, Heredia-Pi I, Andrade-Romo Z, Alcalde-Rabanal J, Bravo L, Jurkiewicz L, Darney BG. Evaluating process fidelity during the implementation of Group Antenatal Care in Mexico. BMC health services research. 2020 Dec;20:1-8. | Mexico | Descriptive study of model fidelity after implementation. Refers to existing theories and examines fidelity to published features of the Centering model, using a checklist. Overall fidelity was high but with some variability.  No relevant theory data included. |
| Gareau S, Lopez-De Fede A, Loudermilk BL, Cummings TH, Hardin JW, Picklesimer AH, Crouch E, Covington-Kolb S. Group prenatal care results in Medicaid savings with better outcomes: a propensity score analysis of CenteringPregnancy participation in South Carolina. Maternal and child health journal. 2016 Jul;20:1384-93. | USA | Retrospective cohort study of Centering Pregnancy in Medicare in US found cost savings relating to prevention of adverse birth outcomes.  No relevant theory data included. |
| Gennaro S, Melnyk BM, O'Connor C, Gibeau AM, Nadel E. Improving prenatal care for minority women. MCN. The American journal of maternal child nursing. 2016 May;41(3):147. | USA | Discussion article on range of models of care which notes that group care may address health behaviours relating to physical and mental health, which may ‘decrease some of the disparities in birth outcomes that are well documented between minority and majority women, as minority women are known to experience increased levels of stress, anxiety, and depression.’ |
| Gholipour K, Tabrizi JS, Asghari Jafarabadi M, Iezadi S, Mardi A. Effects of customer self-audit on the quality of maternity care in Tabriz: a cluster-randomized controlled trial. PLoS One. 2018 Oct 11;13(10):e0203255. | Iran | Customer self-audit of CenteringPregnancy® or usual care within the context of a RCT. The intervention group scored higher on the Service Quality aspects confidentiality, communication, autonomy, availability of support group, dignity, safety, prevention, and accessibility.  No account of the model itself apart from referring to existing evidence on satisfaction and potential empowerment. Findings suggest it may enhance empowerment for this clientele. |
| Grant JH, Handwerk K, Baker K, Milling V, Barlow S, Vladutiu CJ. Implementing Group Prenatal Care in Southwest Georgia Through Public–Private Partnerships. Maternal and Child Health Journal. 2018 Nov;22:1535-42. | USA | Describes the successful implementation of CenteringPregnancy in a public health setting with no prior prenatal services; assesses the program’s first 5-year perinatal outcomes; and discusses several key lessons learned using review of medical records. Refers to previous work showing improved satisfaction and uptake of care but no further information on model. Reports success in implementing and increasing attendance rates. |
| Heberlein EC, Frongillo EA, Picklesimer AH, Covington-Kolb S. Effects of group prenatal care on food insecurity during late pregnancy and early postpartum. Maternal and child health journal. 2016 May;20:1014-24.  Heberlein EC, Picklesimer AH, Billings DL, Covington‐Kolb S, Farber N, Frongillo EA. Qualitative comparison of women's perspectives on the functions and benefits of group and individual prenatal care. Journal of midwifery & women's health. 2016 Mar;61(2):224-34.  Heberlein EC, Picklesimer AH, Billings DL, Covington-Kolb S, Farber N, Frongillo EA. The comparative effects of group prenatal care on psychosocial outcomes. Archives of women's mental health. 2016 Apr;19:259-69. | USA | Further papers from a doctoral study included in the original review: full data of theories obtained from PhD thesis in original analysis. The additional papers corroborate earlier reports of positive impact on stress, confidence, knowledge, motivation, informed decision making, and health care engagement rather than adding new theoretical information or findings |
| Heredia‐Pi IB, Fuentes‐Rivera E, Andrade‐Romo Z, Bravo Bolaños Cacho MD, Alcalde‐Rabanal J, Jurkiewicz L, Darney BG. The Mexican experience adapting CenteringPregnancy: lessons learned in a publicly funded health care system serving vulnerable women. Journal of midwifery & women's health. 2018 Sep;63(5):602-10. | Mexico | Implementation focused account of adapting the standard Centering Model for the Mexican context. Basic account of Centering and refers to the challenge of moving towards a more facilitative model in a hierarchical medical context. |
| Hetherington E, Tough S, McNeil D, Bayrampour H, Metcalfe A. Vulnerable women’s perceptions of individual versus group prenatal care: results of a cross-sectional survey. Maternal and Child Health Journal. 2018 Nov;22:1632-8. | Canada | This survey aimed to assess patient experience among vulnerable women in group prenatal care compared to individual care. Reports that women felt better informed and that providers were more interested in them. |
| Hodgson ZG, Saxell L, Christians JK. An evaluation of Interprofessional group antenatal care: a prospective comparative study. BMC Pregnancy and childbirth. 2017 Dec;17:1-9. | Canada | Cohort study and survey compares outcomes in women receiving interprofessional group perinatal care ‘Connecting Pregnancy’ versus interprofessional individual care (nurses, midwives and physicians, plus sometimes guests such as doulas). Group care considered similar in this programme but with potential to enhance quality of information. |
| Ickovics JR, Earnshaw V, Lewis JB, Kershaw TS, Magriples U, Stasko E, Rising SS, Cassells A, Cunningham S, Bernstein P, Tobin JN. Cluster randomized controlled trial of group prenatal care: perinatal outcomes among adolescents in New York City health centers. American journal of public health. 2016 Feb;106(2):359-65. | USA | RCT of Perinatal outcomes. Women at intervention sites were significantly less likely to have infants small for gestational age; women with more group visits had improvements in gestational age, birth weight, days in neonatal intensive care unit, rapid repeat pregnancy, condom use, and unprotected sex with no associated risks. No additional data on the model or theory of effect but identified improvements for adolescents. |
| Jensen MN, Fage-Butler AM. Antenatal group consultations: Facilitating patient-patient education. Patient Education and Counseling. 2016 Dec 1;99(12):1999-2004. | Denmark | Qualitative study focused on understanding whether and how peer learning is facilitated in group settings; peer learning conceptualised as patient-patient education. Main focus of gANC in Denmark is described as health promotion and it is hypothesised that peer-learning enhances this, via practical, experiential and emotional knowledge. ‘Groupness’ of the group was considered to enhance peer learning and did not depend on homogeneity because of shared experience of pregnancy/birth, along with facilitative skills of the midwife. |
| Jolivet RR, Uttekar BV, O’Connor M, Lakhwani K, Sharma J, Wegner MN. Exploring perceptions of group antenatal Care in Urban India: results of a feasibility study. Reproductive health. 2018 Dec;15(1):1-1. | India | Focused on potential implementation of a generic model of GANC in India, combined from Centering Pregnancy and the Home-based Lifesaving Skills Progamme, aligned with local guidelines. No detail of description of theories. Gathered provider, women and families’ views about acceptability and feasibility, based on a single demonstration in 3 health-service settings. GANC was seen as having potential to improve experiences of care, empower women to become more active partners and participants in their care, through self-assessment, active learning and peer support. |
| Kabue MM, Grenier L, Suhowatsky S, Oyetunji J, Ugwa E, Onguti B, Omanga E, Gichangi A, Wambua J, Waka C, Enne J. Group versus individual antenatal and first year postpartum care: Study protocol for a multi-country cluster randomized controlled trial in Kenya and Nigeria. Gates Open Research. 2018;2. | Kenya and Nigeria | Protocol for a RCT comparing GANC and individual care in two African countries. Refers only to general aim of improving care uptake and outcomes and to test the model in a low-income country context. |
| Kania-Richmond A, Hetherington E, McNeil D, Bayrampour H, Tough S, Metcalfe A. The impact of introducing centering pregnancy in a community health setting: a qualitative study of experiences and perspectives of health center clinical and support staff. Maternal and Child Health Journal. 2017 Jun;21:1327-35. | Canada | Focused on staff experience and perceptions of integration in an existing clinic setting, challenges and opportunities. Centering Pregnancy; No specific focus on the model itself. |
| Kearney L, Kynn M, Craswell A, Reed R. The relationship between midwife-led group-based versus conventional antenatal care and mode of birth: a matched cohort study. BMC pregnancy and childbirth. 2017 Dec;17(1):1-7. | Australia | Retrospective matched cohort study focused on clinical birth outcomes. Non-significant association with reduced rate of CS was found. Basic description of the model ‘Expecting and Connecting’ as interactive, less didactic and facilitating midwife continuity of carer antenatally; related theories not discussed but previous evidence of impact was reviewed briefly. |
| Kominiarek MA, Crockett A, Covington-Kolb MS, Simon M, Grobman WA. Association of group prenatal care with gestational weight gain. Obstetrics and gynecology. 2017 Apr;129(4):663. | USA | Retrospective cohort study of association of group prenatal care (Centering Pregnancy) with gestational weight gain. Hypothesises that the focus on skills, social support and self-monitoring may assist weight management. No differences were found but this could have been related to confounding factors. |
| Kweekel L, Gerrits T, Rijnders M, Brown P. The role of trust in centeringpregnancy: building interpersonal trust relationships in group‐based prenatal care in The Netherlands. Birth. 2017 Mar;44(1):41-7. | The Netherlands | Qualitative study with women who had experienced Centering Pregnancy. Concept of trust was identified as a key mechanism enhancing the group processes of social support and sharing. ‘Trust facilitated social support which in turn enabled reassurance and the building of women's self‐confidence.’  Examines the mechanisms that create trusting relationships within CP to better understand CP outcomes and effectiveness.  Enhances existing analysis. |
| Little, S. H., & Fetters, M. D. (2019). Transcultural modifications of a Japanese language group prenatal care program for transcultural adaptation. Journal of Transcultural Nursing, 30(2), 106-114. | Japan | Account of the transcultural adaptation of a group prenatal care program for Japanese women. Refers only to existing evidence on benefits. Adaptations were mainly in detail of discussion content apart from adaptations to increase privacy: use of a divider screen and avoidance of discussion of sensitive topics in group visits; reduction in number of group visits from 10 to 6. |
| Lori JR, Ofosu-Darkwah H, Boyd CJ, Banerjee T, Adanu RM. Improving health literacy through group antenatal care: a prospective cohort study. BMC Pregnancy and Childbirth. 2017 Dec;17(1):1-9. | Ghana | Cohort study to assess impact of group care on health literacy, conceptualised as ability to understand and act on health messages from professionals, especially relating to risk warning signs and breastfeeding. Time and an interactive and patient-centred approach to information giving were hypothesised as contributors to enhanced literacy. |
| Lori JR, Chuey M, Munro-Kramer ML, Ofosu-Darkwah H, Adanu RM. Increasing postpartum family planning uptake through group antenatal care: a longitudinal prospective cohort design. Reproductive health. 2018 Dec;15:1-8. | Ghana | Prospective cohort study. Women were followed for one-year postpartum to examine the uptake and continuation of family planning following enrolment in group versus individual ANC. Family Planning uptake and breastfeeding rates found to be improved. Hypothesis based on view that in GANC women would have more time to gain and process information about postnatal health issues. |
| Liu R, Chao MT, Jostad-Laswell A, Duncan LG. Does CenteringPregnancy group prenatal care affect the birth experience of underserved women? A mixed methods analysis. Journal of immigrant and minority health. 2017 Apr;19:415-22. | USA | Qualitative study focused on birth experiences of immigrant and minority women and how CenteringPregnancy may have influenced these. Model is examined through reference to existing CP literature although they highlight the concept of self-efficacy as being central. A mindfulness approach was incorporated but this is not explained. Centering was reported as providing women with pain coping skills and knowledge to advocate for themselves. The few women who were attended by a Centering facilitator valued this continuity highly. |
| Magriples U, Boynton MH, Kershaw TS, Lewis J, Rising SS, Tobin JN, Epel E, Ickovics JR. The impact of group prenatal care on pregnancy and postpartum weight trajectories. American journal of obstetrics and gynecology. 2015 Nov 1;213(5):688-e1. | USA | Secondary analysis of a cluster RCT; medical record review and structured interviews to evaluate the weight change trajectories in the control and intervention groups. They note lack of evidence of benefit from direct interventions and hypothesise that the group model may enhance weight management through discussion, skills building and stress reduction. |
| McDonald SD, Sword W, Eryuzlu LN, Neupane B, Beyene J, Biringer AB. Why are half of women interested in participating in group prenatal care?. Maternal and child health journal. 2016 Jan;20:97-105. | Canada | Self-administered questionnaire. Characteristics of women who accept to participate in gANC and why they do. Refers to existing evidence on outcomes with no further information. |
| Patil CL, Klima CS, Leshabari SC, Steffen AD, Pauls H, McGown M, Norr KF. Randomized controlled pilot of a group antenatal care model and the sociodemographic factors associated with pregnancy-related empowerment in sub-Saharan Africa. BMC pregnancy and childbirth. 2017 Nov;17(2):1-0.  Patil CL, Klima CS, Steffen AD, Leshabari SC, Pauls H, Norr KF. Implementation challenges and outcomes of a randomized controlled pilot study of a group prenatal care model in Malawi and Tanzania. International Journal of Gynecology & Obstetrics. 2017 Dec;139(3):290-6. | Malawi and Tanzania | Pilot RCT of Group ANC to test hypothesis that the model increases empowerment of pregnant women. Variable findings with indication that empowerment was increased for some groups (Muslim women) and settings (Malawi), but not all. Empowerment was conceptualised as ability of individuals to ‘improve capacities, to critically evaluate situations and to take actions to improve those situations’ and understood as positively associated with uptake of maternity and reproductive services and improved infant outcomes. Empowerment was also seen as related to sense of control and ability to adopt healthy behaviours. The concept was explicitly drawn from Bandura’s self-efficacy theory, feminist and social theory and seen as supported by community building, continuity of carer and building self-care skills in gANC.  Linked study report focused on implementation challenges and pilot trial outcomes. |
| Riggs E, Muyeen S, Brown S, Dawson W, Petschel P, Tardiff W, Norman F, Vanpraag D, Szwarc J, Yelland J. Cultural safety and belonging for refugee background women attending group pregnancy care: an Australian qualitative study. Birth. 2017 Jun;44(2):145-52. | Australia | ‘Women reported feeling empowered and confident through learning about pregnancy and childbirth in the group setting. The collective sharing of stories in the facilitated environment allowed women to feel prepared, confident and reassured, with the greatest benefits coming from storytelling with peers, and developing trusting relationships with a team of professionals, with whom women were able to communicate in their own language. Women also discussed the pivotal role of the bicultural worker in the multidisciplinary care team.’ |
| Rijnders M, Jans S, Aalhuizen I, Detmar S, Crone M. Women‐centered care: Implementation of CenteringPregnancy® in The Netherlands. Birth. 2019 Sep;46(3):450-60. | The Netherlands | Retrospective cohort study (n = 2318) and survey to investigate outcome differences between CP and traditional individual prenatal care. Focused on feasibility of implementation but refers to empowerment and satisfaction with care in a context of low satisfaction among Dutch women. |
| Sayinzoga F, Lundeen T, Gakwerere M, Manzi E, Nsaba YD, Umuziga MP, Kalisa IR, Musange SF, Walker D. Use of a facilitated group process to design and implement a group antenatal and postnatal care program in Rwanda. Journal of Midwifery & Women's Health. 2018 Sep;63(5):593-601. | Rwanda | Describes the process of adapting a Centering-based group model to the Rwandan context and development of an implementation plan. Refers to existing evidence on potential benefits. Peer (equal) approach to the group was seen as a 1^st^ principle. No further information is provided on the potential mechanisms or principles. The article is useful primarily in setting out a consultation process for adaptation and implementation. |
| Tilden EL, Emeis CL, Caughey AB, Weinstein SR, Futernick SB, Lee CS. The influence of group versus individual prenatal care on phase of labor at hospital admission. Journal of Midwifery & Women's Health. 2016 Jul;61(4):427-34. | USA | Retrospective case control study of influence of Group Versus Individual Prenatal Care on Phase of Labor at Hospital Admission. Hypothesis that this may reduce admission in latent phase of labour as a result of improved information and understanding about labour. Significant differences were found with women who received GANC more likely to be admitted in active labour. |
| Trotman G, Chhatre G, Darolia R, Tefera E, Damle L, Gomez-Lobo V. The effect of centering pregnancy versus traditional prenatal care models on improved adolescent health behaviors in the perinatal period. Journal of pediatric and adolescent gynecology. 2015 Oct 1;28(5):395-401. | USA | Known features of this model - education, group discussion, and social support considered helpful to address needs of adolescents for support, to reduce isolation and increase healthy behaviours.  Improved compliance with prenatal visits, uptake of LARC methods, adequate weight gain, and increased rates of breastfeeding were identified and seen as associated with improved healthy habits via psychosocial support and education. |
| Tubay AT, Mansalis KA, Simpson MJ, Armitage NH, Briscoe G, Potts V. The effects of group prenatal care on infant birthweight and maternal well-being: a randomized controlled trial. Military medicine. 2019 May 1;184(5-6):e440-6. | USA | RCT with women from the military receiving either traditional one-on-one prenatal care or group prenatal care. Focused on clinical outcomes and satisfaction with care. No differences were found apart from improvement in births at appropriate gestational age. Refers to existing theories of the mechanism of effect as peer support and self-management strategies that mitigate stress and reduce high-risk behaviours, and also through reducing isolation, which they see as particularly relevant for a military population. |
| Walton RB, Shaffer S, Heaton J. Group prenatal care outcomes in a military population: a retrospective cohort study. Military medicine. 2015 Jul 1;180(7):825-9. | USA | Retrospective Cohort Study of outcomes in a military population. No significant differences in outcomes were found, which they attributed to small study size. Centering Pregnancy referred to as an integrative approach, combining education, peer support, and family members that has been associated with improved outcomes in some studies. No further information given on the theory. |
| Yorga KD, Sheeder JL. Which pregnant adolescents would be interested in group-based care, and why?. Journal of Pediatric and Adolescent Gynecology. 2015 Dec 1;28(6):508-15. | USA | Study focused on factors that influence pregnant adolescents’ interest in participating in gANC or not. Key reasons to participate were identified as to belong to a peer group, to receive additional education and support and to have fun. Structured survey including some open-response questions. Hypotheses discussed were that the approach may reduce loneliness and isolation for pregnant adolescents, and that it may provide an environment of positive peer support which also supports increase in knowledge, which in turn may enhance healthy behaviours. |
| Zorrilla CD, Sánchez I, Mosquera AM, Sierra D, Pérez LA, Rabionet S, Rivera-Viñas J. Improved infant outcomes with group prenatal care in Puerto Rico. Source journal of obstetrics and gynaecology. 2017;1(1). | Puerto Rico | Retrospective chart review looking at outcomes in Puerto Rican population. Improvements were found in birthweight and gestational age. No information given about rationale for care except that the model has been found to improve uptake and outcomes in other settings. |
